# Supplementary material for: Experimental verification of electro-refractive phase modulation in graphene
Source: Sci Rep. 2015 Jun 10;5:10967. doi: 10.1038/srep10967 (PMC4462031; doi:10.1038/srep10967)
Supplement: Supplementary Information [file srep10967-s1.doc]

Experimental verification of electro-refractive phase modulation in graphene

Muhammad Mohsin*^,1^, Daniel Neumaier^1^, Daniel Schall^1^, Martin Otto^1^, Christopher Matheisen^1^, Anna Lena Giesecke^1^, Abhay A. Sagade^1^ & Heinrich Kurz^1^

^1^Advanced Microelectronic Center Aachen (AMICA), Applied Micro and Optoelectronic (AMO) GmbH, Otto-Blumenthalstr. 25, 52074 Aachen, Germany

* Correspondence and requests for materials should be addressed to M.M. (mohsin@amo.de)

**Supplementary information**

**Variation in wavelength shifts at different minima**

Only one inset is shown in Fig. 2b in manuscript so as to give a general idea of the main findings. All minima show an almost identical shift as is depicted in Fig. S1.

The index modulation, as shown in Fig. 2c, is extracted from one transmission minima around 1551 nm (indicated as 4^th^ resonance in Fig. S1). This was chosen as the change in absorption of the electro-absorption was measured at 1550 nm. However, Fig S2c graphically shows the variation in index modulation for different minima for two mentioned voltages of -40V and +40V. It is obvious that the variation in index modulation at different minima is less than 10% about its mean value. This is expected as the measurements were done in air at room temperature

| 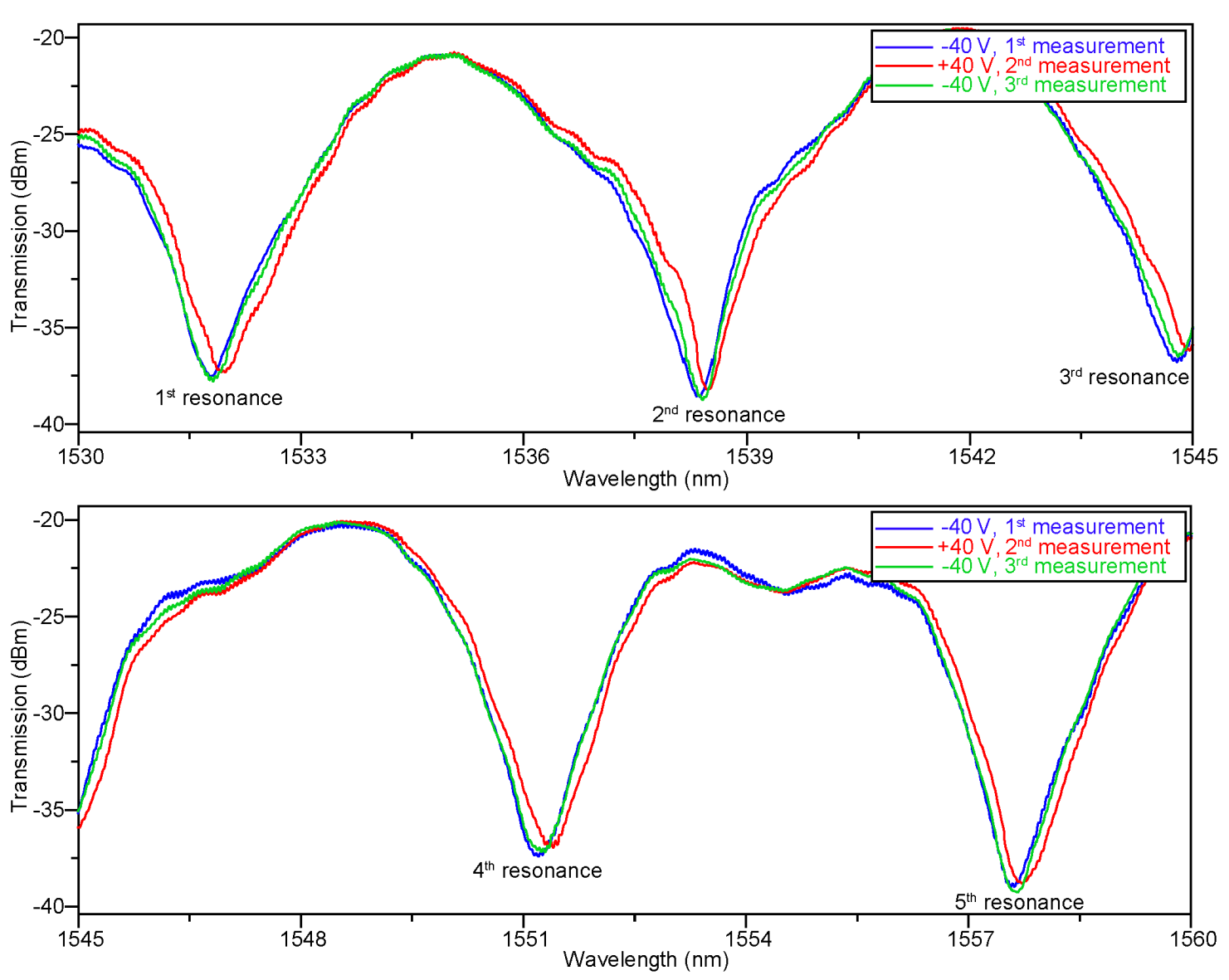 |
| --- |
| **Figure S1.** Measured transmission spectra for two different voltages. The wavelength shift is observable at all resonances. |

| 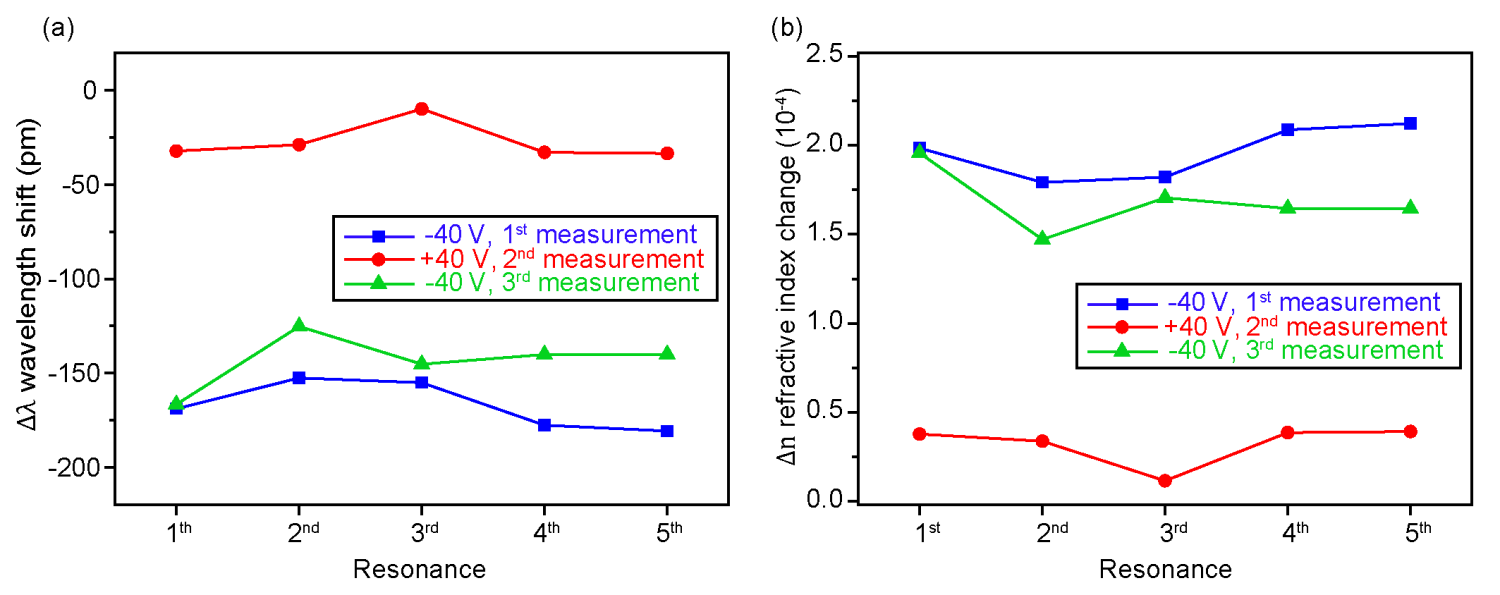 |
| --- |
| **Figure S2. (a)**Variation in wavelength shifts and **(b)** refractive index modulation at different resonances. The values are depicted taking +30V as the reference measurement since the effective refractive index was minimal at +30V as shown in Fig 2c. |
